# Factors affecting the risk of gender-based violence among 2SLGBTQIA+ adolescents and youth: a scoping review of climate change-related vulnerabilities

**DOI:** 10.3389/fsoc.2025.1541039

**Published:** 2025-02-12

**Authors:** Szymon Parzniewski, Xing Luo, Siyu Ru, Nevcihan Ozbilge, Kyle Breen, Haorui Wu

**Affiliations:** ^1^School of Social Work, Dalhousie University, Halifax, NS, Canada; ^2^Department of Sociology, University of Saskatchewan, Saskatoon, SK, Canada; ^3^Department of Social Sciences, Texas A&M International University, Laredo, TX, United States

**Keywords:** 2SLGBTQIA+ youth, 2SLGBTQIA+ adolescents, climate vulnerability, 2SLGBTQIA+ gender-based violence, resilience, adolescent, youth, literature review

## Abstract

Gender-based violence (GBV) is a pervasive and growing issue that affects diverse populations worldwide. This study aimed to synthesize the factors affecting the risk of GBV among 2SLGBTQIA+ adolescents and youth. A scoping review was conducted using international databases (Web of Science, Scopus, Proquest, PubMed, Embase, EBSCOhost) since 2009. The studies were independently appraised by two reviewers guided by the PRISMA approach. Ninety-nine articles focusing on the 2SLGBTQIA+ community, GBV, and adolescents or youth were included for the review. The factors affecting the risk of GBV among 2SLGBTQIA+ adolescents and youth were categorized into (1) Wellbeing and mental health; (2) Disparities compared to cisgender youth; (3) Perpetration and victimization; (4) Different types of violence; and (5) Differences among 2SLGBTQIA+ subgroups. The studies further showed relevance to climate-related stressors such as displacement and resource scarcity, and how those can further amplify the vulnerabilities of 2SLGBTQIA+ youth to GBV. The findings revealed the necessity for multi-level strategies that account for the compounded risks faced by 2SLGBTQIA+ adolescents and youth, including those introduced by environmental crises. These five factors related to GBV among 2SLGBTQIA+ adolescents and youth should be considered by social work professionals when working with at-risk populations. Due to limited robust evidence (from countries outside the US) and the diverse contexts of the selected studies, future research is needed to minimize GBV among 2SLGBTQIA+ adolescents and youth, especially as climate change introduces new layers of vulnerability.

**Systematic review registration:**

10.37766/inplasy2024.4.0008

## 1 Introduction

Gender-based violence (GBV) is a pervasive and growing issue that affects diverse populations worldwide, with some groups experiencing significantly higher risks (Dominey-Howes et al., 2013). Among the most vulnerable are youth and adolescents, particularly 2SLGBTQIA+^1^ individuals, who encounter increased exposure to violence due to intersecting factors such as gender identity, sexual orientation, and age (Mann et al., 2024). These vulnerabilities can be further exacerbated by a range of external factors, such as environmental displacement, resource scarcity, and societal disruptions often heightening the risks of violence and discrimination faced by marginalized groups, including 2SLGBTQIA+ adolescents and youth (henceforth referred to as “2SLGBTQIA+ youth”). As the impacts of various climate-related hazards continue to intensify, vulnerable populations are disproportionately affected, particularly those who already face systemic discrimination and social marginalization (Simmonds et al., 2022). For 2SLGBTQIA+ youth and other marginalized groups, the intersection of environmental disruption and social inequality creates compounded risks and challenges (Goldsmith et al., 2022). Climate change is not just an environmental crisis but a social one, amplifying existing inequalities (Thomas et al., 2019).

Climate change exacerbates vulnerabilities for 2SLGBTQIA+ youth through economic instability (Goldsmith and Bell, 2022). Climate change intensifies extreme weather events such as hurricanes, droughts, and floods, causing widespread disruption to livelihoods. Many 2SLGBTQIA+ youth already face systemic barriers to stable employment due to discrimination and stigma (Kilpatrick et al., 2024). When livelihoods in climate-sensitive industries, such as agriculture, become precarious, this economic instability disproportionately affects those who are already marginalized. Economic stress can lead to financial dependency on abusive relationships, heightening the risk of GBV (van Daalen et al., 2022).

Furthermore, for 2SLGBTQIA+ youth, these impacts can include being disproportionately affected by housing insecurity, increased poverty, and limited access to inclusive emergency services (Gorman-Murray et al., 2014). Social and cultural norms that perpetuate discrimination further intersect with climate change impacts, creating compounding challenges for 2SLGBTQIA+ youth. Displacement caused by extreme weather events often forces individuals into temporary shelters or informal settlements (Yamashita et al., 2017), putting them at greater risk of harassment or neglect during periods of crisis. In these environments, a lack of privacy, security, and inclusivity increases exposure to physical, sexual, and psychological violence (Allen et al., 2024). These risks are especially pronounced in contexts where cultural norms marginalize non-heteronormative identities, leaving 2SLGBTQIA+ individuals unprotected by law enforcement or support services.

Additionally, limited climate change literacy in vulnerable regions compounds these challenges (Nahid and Pain, 2021). While many individuals in these regions perceive worsening weather patterns, they often do not associate these changes with climate change. This lack of awareness undermines efforts to build community resilience, as communities are less equipped to recognize and address the disproportionate impacts of climate change on marginalized groups. For 2SLGBTQIA+ youth, whose experiences often go unacknowledged in mainstream discourse, this gap in understanding further entrenches their exclusion (Whitley and Bowers, 2023). Incorporating climate change education into GBV interventions could empower communities to address these intersecting challenges, fostering resilience at both individual and collective levels.

Despite growing awareness of the severe impacts associated with GBV, there remains a substantial gap in the literature addressing the specific challenges faced by 2SLGBTQIA+ youth, particularly regarding efforts to minimize their exposure to such violence in the context of both social and environmental crises. This gap contributes to the ongoing vulnerability of this population, often leaving many without adequate protection or support.

This literature review aims to provide a comprehensive analysis of existing research on GBV among 2SLGBTQIA+ youth. Although these interconnected issues have been documented (Atteberry-Ash et al., 2019), the review reveals that the factors which exacerbate or mitigate the risk of GBV for this population are underexplored, particularly in disaster-prone environments. Consequently, current responses are often insufficient, failing to address the complex needs and experiences of 2SLGBTQIA+ youth, which perpetuates cycles of harm and marginalization.

By synthesizing the available literature, this review seeks to illuminate the implications of GBV for 2SLGBTQIA+ youth, including its impact on physical and mental health, educational attainment, and the heightened vulnerability they experience during climate-related disasters. It emphasizes the need for research that focuses on strategies to reduce GBV exposure and outlines gaps in existing approaches, particularly in the context of global climate challenges. Through a critical examination of the literature, this article aims to advocate for more effective and targeted interventions, ultimately working toward creating safer environments and supportive systems for all youth, irrespective of their gender identity, sexual orientation, or the environmental contexts.

## 2 Methods

This review was guided by the question, ‘what factors affect the risk of GBV among 2SLGBTQIA+ adolescent and youth?' For the purpose of this study, a “scoping review” is defined as a systematic and comprehensive approach to mapping literature aimed at identifying key concepts, research gaps, and evidence relevant to informing practice, policy, and future research (Daudt et al., 2013). This scoping review was guided by the Preferred Reporting Items for Systematic Review and Meta-Analysis (PRISMA) approach (Page et al., 2021; Reedman et al., 2024) and followed three steps of literature searching, screening, and data analysis.

### 2.1 Searching strategies and databases

We searched six databases—Web of Science, Scopus, Proquest, PubMed, Embase and EBSCOhost—to identify literature. These databases provide access to full-text content from worldwide academic sources. The preliminary list of keywords was generated by reviewing the research question, objectives, and relevant literature in the field. Synonyms, alternative spellings, and variations (e.g., “LGBTQ2SIA+” vs. “2SLGBTQI+”) were included to ensure comprehensiveness. Input was sought from subject-matter experts to identify discipline-specific terminology and emerging trends. In addition, keywords and index terms from highly relevant articles were reviewed to identify additional search terms. The search strategy was pilot tested to ensure its effectiveness. This included two elements. First, initial searches were conducted and retrieved results were reviewed to ensure the inclusion of key studies. Second, keywords were refined based on pilot results, adding terms for missing concepts and removing redundant or overly complex terms.

Finally, we created three groups of keywords to search for literature on 2SLGBTQI+, GBV and adolescent (Table 1). The Boolean operator “OR” was used within each keyword group, and “AND” was used between the groups to refine the search. To further narrow the results, we focused on academic journals published from 2009 to 2024 and written in English. The whole data set was uploaded to COVIDENCE, a web-based platform that facilitates the conduction of a comprehensive literature review (Page et al., 2021).

**Table 1:** Database search keywords.

**Keyword groups**	**Keywords**
2SLGBTQI+	“Lesbian” OR “Gay” OR “Bisexual” OR “Transgender” OR “Queer” OR “Intersex” OR “Asexual” OR “Sexual orientation” OR “Cisgender” OR “Non-binary” OR “Transphobia” OR “LGBT” OR “LGBTQ” OR “LGBTQA+” OR “LGBTQ2” OR “LGBTQ2SIA+” OR “LGBTQIA2S+” OR “2SLGBTQI+”
GBV	“Gender-based violence” OR “Gender violence” OR “Sexual abuse” OR “Physical abuse” OR “Emotional violence” OR “Sexual harassment” OR “Dating violence” OR “Intimate partner violence”
Youth	“Adolescent” OR “Youth”

### 2.2 Screening

**Figure 1:**
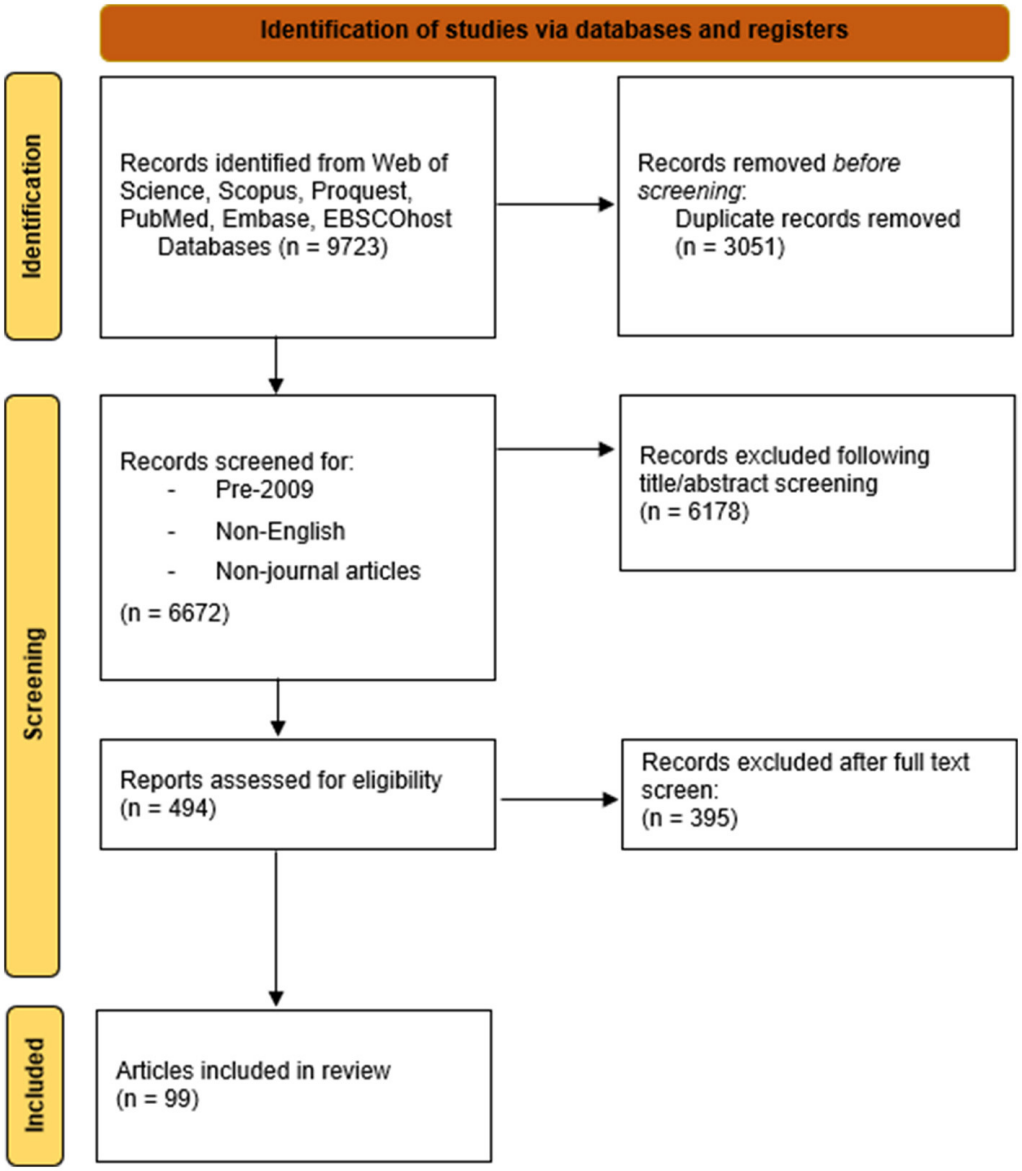
PRISMA chart.

This review encompassed 12,850 (*n* = 9,723) studies in total, following the removal of duplicates and those marked ineligible by automation tools, the remaining studies (*n* = 6,672) were assessed for eligibility by four researchers, with 99 studies (*n* = 99) meeting the final criteria (Figure 1). All of the studies that met the final inclusion criteria for eligibility explored 2SLGBTQIA+ youth experiences with GBV. The general inclusion criteria consisted of: (1) publication year: All included studies were published between 2009 and 2024 (2) studies focusing on 2slgbtqia+ adolescent; 2slgbtqia+ youth; 2slgbtqia+ high school student; same-sex partner adolescent or youth. The general exclusion criteria consisted of: (1) 2slgbtqia+ adult or 2slgbtqia+ college/university students (Table 2).

**Table 2:** Inclusion and exclusion criteria.

**Inclusion criteria**	**Exclusion criteria**
■2slgbtqia+ violence victimization Adolescent ■2slgbtqia+ & GBV Adolescent ■2slgbtqia+ & GBV-health-related impacts ■2slgbtqia+ inclusive GBV & adolescent/youth support program Sexual harassment of ■2slgbtqia+ adolescent Dating violence of ■2slgbtqia+ adolescent Family violence of ■2slgbtqia+ adolescent Psychological distress of ■2slgbtqia+ adolescent Intimate partner violence of ■2slgbtqia+ adolescent	■2slgbtqia+ adult and GBV ■2slgbtqia+ childhood violence only ■2slgbtqia+ adult and mental health Non-GBV-related violence Non-GBV-related family violence Cisgender adolescent and GBV Cisgender childhood violence Primary focus on HIV

Among the 99 included studies, 60% being most recently published within the past five years (*n* = 59/99). The majority of all studies (*n* = 77/99) studies were conducted in the United States of America, with the remaining studies from Australia (*n* = 2/99), Belgium (*n* = 2/99), Brazil (*n* = 2/99), Canada (*n* = 5/99), China (*n* = 1/99), Finland (*n* = 3/99), Israel (*n* = 1/99), Mexico (*n* = 1/99), Netherlands (*n* = 1/99), Spain (*n* = 2/99), Thailand (*n* = 3/99).

### 2.3 Data analysis

An inductive thematic analysis was conducted for data analysis (Caulfield, 2019). Two student research assistants were trained to independently reviewed and coded the articles using Excel spreadsheets. The research team then compared the codes and resolved any conflicts through team discussions. Ultimately, the research team formulated overarching themes and subthemes The resulting themes have been categorized to highlight the findings from the research and gain a more nuanced and in-depth understanding of the topic of 2SLGBTQIA+ adolescent and youth experiences with Gender Based Violence (GBV) (Table 3).

**Table 3:** Themes identified from thematic analysis.

**Theme**	**Description**	**Article examples**
Wellbeing and mental health	Victimization of (GBV) negatively impacted the mental health and general wellbeing of 2SLGBTQIA+ youth	Atteberry-Ash et al., 2019; Bouris et al., 2016; Dunn et al., 2017; Gámez-Guadix and Incera, 2021; de Medeiros et al., 2023; Zeglin et al., 2020
Disparities compared to cisgender youth	2SLGBTQIA+ youth generally are more likely to experience GBV and GBV-related adverse mental health outcomes compared to heterosexual and cisgender youth	Martin-Storey et al., 2021; Johns et al., 2018; Rostad et al., 2020; Sabina et al., 2022; Zeglin et al., 2020
Perpetration and victimization	Risk factors for violence perpetration and victimization include individual, behavioral, mental, parental, societal, sociodemographic, and social service-based factors	Adhia et al., 2021; Chan and Lam, 2023; Langenderfer-Magruder et al., 2016; MacAulay et al., 2022; Stroem et al., 2021; Whitton et al., 2019a
Different types of violence	2SLGBTQIA+ youth experienced various types of GBV and had different perceptions and help-seeking behaviors	Brewer et al., 2024; Kaufman and Baams, 2022; Nath et al., 2022; Price et al., 2023; Wright et al., 2023
Differences among 2SLGBTQIA+ subgroups	Demographic factors influences 2SLGBTQIA+ youth's experiences in violence perpetration and victimization	Kosciw et al., 2012; Mitchell et al., 2014; Strauss et al., 2020; Taylor et al., 2021; Whitton et al., 2019a

## 3 Results

### 3.1 Theme 1: wellbeing and mental health outcomes

The reviewed studies demonstrate that victimization due to exposure to intimate partner violence (IPV), dating violence, family violence, school violence, bullying, sexual harassment, and sexual assault among 2SLGBTQIA+ youth is associated with a higher risk of adverse mental health outcomes, such as depression, anxiety, suicidal ideation, and substance use (Atteberry-Ash et al., 2019; Bouris et al., 2016; Burgwal et al., 2023; Do et al., 2020; Dunn et al., 2017; Edwards, 2018; Espelage et al., 2018; Fish et al., 2023; Gámez-Guadix and Incera, 2021; Johns et al., 2018; Kiekens et al., 2022; Lian et al., 2022; Marx et al., 2021; Meadows et al., 2022; de Medeiros et al., 2023; Mintz et al., 2021; Mitchell et al., 2014, 2023; Nydegger et al., 2020; Patrick et al., 2013; Price et al., 2023; Sabina et al., 2022; Smith et al., 2020; Strauss et al., 2020; Taber et al., 2023; Walls et al., 2019; Wichaidit et al., 2021, 2023; Zeglin et al., 2020). These mental health risks can be further exacerbated by climate change-related vulnerabilities, as environmental crises may increase the stress and instability experienced by already marginalized 2SLGBTQIA+ youth. Victimization among 2SLGBTQIA+ youth is associated with higher levels of substance use (Johns et al., 2018; Kiekens et al., 2022; Wichaidit et al., 2021, 2023). For instance, substance use among victimized 2SLGBTQIA+ youth is reported to be elevated (Johns et al., 2018), and individuals identifying as 2SLGBTQIA+ have a higher prevalence of alcohol use (Wichaidit et al., 2023). In the context of climate-related displacement or instability, these behaviors may increase as coping mechanisms in response to heightened stress and reduced access to support systems.

Moreover, victimization among 2SLGBTQIA+ individuals is associated with a higher risk of depression, depressed mood, and depressive symptoms (Mitchell et al., 2023; Patrick et al., 2013; Walls et al., 2019; Wichaidit et al., 2023), and anxiety (Gámez-Guadix and Incera, 2021; Price et al., 2023; Sabina et al., 2022). Suicidality or suicidal ideation is related to higher rates of victimization among 2SLGBTQIA+ youth (Bouris et al., 2016; Dunn et al., 2017; Espelage et al., 2018; Marx et al., 2021; Nydegger et al., 2020). For example, 2SLGBTQIA+ youth who have experienced school violence and crime are at higher risk of suicidality (Espelage et al., 2018), and sexual victimization is a predictor of suicidal ideation among transgender and gender-nonconforming youth (Marx et al., 2021). When compounded by the stresses of climate change, such as displacement, loss of community, and scarcity of resources, the risk of mental health decline, including suicidality, may become even more severe for these vulnerable populations.

The higher prevalence of adverse mental health outcomes among victimized 2SLGBTQIA+ youth is associated with environmental factors such as school belonging, family support, social support, and sociodemographic variables (Hazelwood, 2023; Marx et al., 2021; Mintz et al., 2021; Mitchell et al., 2014; Smith et al., 2020). For instance, family support serves as a protective factor against depression among researched youth (Mintz et al., 2021). Similarly, parental support and school belonging are linked to lower levels of suicidal ideation among transgender or gender-nonconforming youth (Marx et al., 2021). In the wake of climate-related crises, these protective factors become even more crucial, as the stability provided by family and social support can buffer against the compounded effects of victimization and environmental stress.

### 3.2 Theme 2: disparities in mental health and violence experiences between 2SLGBTQIA+ and cisgender, heterosexual youth

2SLGBTQIA+ youth generally face significantly higher levels of violence, including victimization, perpetration, or both, compared to their heterosexual and cisgender peers (Martin-Storey et al., 2021). Studies indicate that 2SLGBTQIA+ youth report higher rates of various forms of violence, including abuse, assault, bullying, harassment, threatening behaviors, dating violence, physical and sexual violence, peer victimization, and intimate partner violence (Edwards, 2018; Hazelwood, 2023; Hequembourg et al., 2020; Kaltiala-Heino et al., 2019; Luo et al., 2014; Mitchell et al., 2023; Norris and Orchowski, 2020; Petit et al., 2020; Rostad et al., 2020; Sabina et al., 2022; Williams and Gutierrez, 2022). 2SLGBTQIA+ youth are significantly more likely to be exposed to various types of violence, such as verbal, physical, cyber, and sexual victimization, compared to cisgender youth (Atteberry-Ash et al., 2019; Dank et al., 2014; Garthe et al., 2021, 2023; Kattari et al., 2021; Norris and Orchowski, 2020; Wichaidit et al., 2023), including monovictimization and polyvictimization (Mitchell et al., 2023; Petit et al., 2020; Schwab-Reese et al., 2021). Similarly, in the context of climate change-related crises, the vulnerability of 2SLGBTQIA+ youth to violence may be further exacerbated, as environmental stressors such as displacement, resource scarcity, and the breakdown of social networks create additional risks of victimization.

Experiences of violence victimization generally increase the likelihood of 2SLGBTQIA+ youth reporting more severe adverse health outcomes and negative health behaviors (Pathela and Schillinger, 2010). 2SLGBTQIA+ dating violence victims reported more severe outcomes than heterosexual dating violence victims, including higher rates of depression, binge drinking, and poorer academic performance (Edwards, 2018). Among students who were recently bullied, 2SLGBTQIA+ boys were more likely to seriously contemplate suicide compared to their heterosexual peers, while 2SLGBTQIA+ girls had a higher risk of depression and suicidal ideation (Dunn et al., 2017). Climate-related stressors, such as displacement or disrupted educational environments, could further exacerbate these mental health challenges, particularly for already marginalized 2SLGBTQIA+ youth. For example, a young transgender person displaced by a hurricane may lose access to an affirming school environment, gender-affirming healthcare, or a local LGBTQIA+ support group, leaving them vulnerable to increased anxiety and depression. If relocated to a community or school that lacks inclusivity or exposes them to discrimination, their sense of safety and belonging can further erode, compounding feelings of rejection and marginalization. The impact of sexual identity and violence victimization on health outcomes has been shown to vary. For instance, the interaction between 2SLGBTQIA+ identity and a history of sexual assault was statistically significant only for boys (Zeglin et al., 2020). Among boys, the impact of a sexual assault history on the likelihood of reporting depression was more pronounced for those identifying as straight compared to those identifying as 2SLGBTQIA+ (Zeglin et al., 2020).

In general, 2SLGBTQIA+ youth are associated with higher risks of depression, anxiety, hostility, and substance use, such as cigarette smoking and illicit drug usage, compared to their heterosexual counterparts (Johns et al., 2018; Rostad et al., 2020; Sabina et al., 2022; Zeglin et al., 2020). Rates of suicidal thoughts and suicide attempts are also higher among 2SLGBTQIA+ youth (Johns et al., 2018; Nydegger et al., 2020). These risks are likely to increase in the context of climate-related crises, as the added stressors of environmental disruption may lead to further declines in mental health and an increased likelihood of harmful coping mechanisms such as substance use. These associations vary by sexual orientation and gender identity. Compared to cisgender youth and transgender girls, transgender boys experience higher risks of depression, suicidality, and a history of alcohol consumption, while transgender girls are less likely to report binge drinking (Wichaidit et al., 2021, 2023). Transgender boys and cisgender 2SLGBTQIA+ girls are more likely to engage in substance use than cisgender heterosexual boys, and they have a greater likelihood of experiencing depressed mood compared to other genders (Mitchell et al., 2023). Transgender and cisgender females are also more likely to have attempted suicide compared to cisgender males (Shilo et al., 2021).

Regarding violence perpetration, 2SLGBTQIA+ youth reported higher rates of perpetration for physical and cyber dating violence and abuse compared to heterosexual, cisgender youth (Dank et al., 2014). Variations in perpetration rates were also observed among 2SLGBTQIA+ youth (Espelage et al., 2022). Gay males were less likely to bully others than heterosexual males, while 2SLGBTQIA+ females, except for lesbians, were more likely to report bullying others than heterosexual females (Berlan et al., 2010). Another study suggests that 2SLGBTQIA+ youth had a similar likelihood of perpetrating sexual violence as cisgender youth, but aggressive behavior was linked to an increased likelihood of sexual violence perpetration among transgender boys and girls, as well as nonbinary youth (Ybarra et al., 2022). In the context of climate-induced social instability, these aggressive behaviors may become more prevalent, as displaced or marginalized youth experience heightened stress and fewer opportunities for positive social engagement (Faas and Parreno, 2024). As for the risk factors associated with perpetration, discrimination and stigma against 2SLGBTQIA+ youth were identified as unique contributors to their involvement in both perpetration and victimization (Nath et al., 2022). However, another study shows that experiencing victimization significantly increased the odds of engaging in violence perpetration for both 2SLGBTQIA+ and heterosexual youth (Messinger et al., 2018). In times of environmental crises, addressing the dual roles of victimization and perpetration in 2SLGBTQIA+ youth becomes even more critical, as both may be exacerbated by the increased stress and disruption brought on by climate change.

### 3.3 Theme 3: risk factors for violence perpetration and victimization

The reviewed studies indicate that violence perpetration and victimization among 2SLGBTQIA+ youth, including sexual orientation harassment, partner violence, dating violence, and relationship abuse, are associated with individual, behavioral, mental, parental, societal, sociodemographic, and social service-based risk factors (Adhia et al., 2021; Chan and Lam, 2023; Dyar et al., 2020; de Castro Jury Arnoud et al., 2024; Goldenberg et al., 2018; Hazelwood, 2023; Hequembourg et al., 2020; Kosciw et al., 2009; Langenderfer-Magruder et al., 2016; MacAulay et al., 2022; Martin-Storey et al., 2021; Marx et al., 2021; Murchison et al., 2019; Rostad et al., 2020; Stroem et al., 2021; Valido et al., 2021; Ybarra et al., 2022; Whitton et al., 2019a). In the context of climate change, these vulnerabilities may be heightened, as environmental crises like displacement and resource scarcity exacerbate the risk factors already affecting 2SLGBTQIA+ youth, particularly those facing instability in housing or access to social services (Yamashita et al., 2017).

Physical and sexual dating or IPV victimization pose a higher risk for 2SLGBTQIA+ youth compared to cisgender youth (Adams et al., 2020; Dank et al., 2014; Hazelwood, 2023; Hequembourg et al., 2020; Martin-Storey, 2015; Martin-Storey et al., 2021; Norris et al., 2022; Whitton et al., 2019a). The increased odds of sexual and physical IPV victimization among 2SLGBTQIA+ youth are associated with having more sexual partners (Whitton et al., 2019a). Substance use is another factor linked to a higher likelihood of experiencing physical IPV (Langenderfer-Magruder et al., 2016; Norris et al., 2022; Reuter et al., 2015; Rostad et al., 2020; Whitton et al., 2019a). Substance use may further escalate in times of climate-related crises, as the stress and instability caused by environmental disruption lead to increased reliance on harmful coping mechanisms. Previous violence exposure and gender stereotyping also contribute to a higher risk of victimization (Langenderfer-Magruder et al., 2016; Taylor et al., 2021). Additionally, studies show that episodes of homelessness are associated with increased partner violence victimization among 2SLGBTQIA+ youth (Langenderfer-Magruder et al., 2016). Climate change may exacerbate homelessness among these vulnerable groups, leading to greater exposure to violence, particularly IPV, as they face limited shelter options and heightened social instability (Yamashita et al., 2017). Moreover, learning experiences and the normative climate in schools are factors related to the elevated risk of sexual violence victimization and perpetration for 2SLGBTQIA+ youth (Chan and Lam, 2023; MacAulay et al., 2022).

Beyond these traditional risk factors, sexual orientation itself is an additional risk factor for higher rates of dating violence victimization (Hazelwood, 2023; Hequembourg et al., 2020; Reuter et al., 2015). As climate-related crises increase societal stress and resource scarcity, the risks associated with sexual orientation and identity may be further amplified, increasing the likelihood of violence victimization. While sexual identity and attractions may not directly cause IPV, sexual behavior is linked to increased risk (Dyar et al., 2020). Regardless of sexual orientation, gender nonconformity is a significant risk factor for IPV (Adhia et al., 2021). Other structural factors, such as a history of incarceration and sex work, along with depressive symptomatology as an intrapersonal factor, are also associated with higher reports of IPV (Goldenberg et al., 2018). In times of environmental disruption, the compounding effects of marginalization—such as a history of homelessness, sex work, or incarceration—can increase the vulnerability of 2SLGBTQIA+ youth to IPV and other forms of violence. Some of the reviewed studies identify protective factors that can reduce the prevalence of violence victimization and perpetration among 2SLGBTQIA+ youth. These include family, peer, and community support, helping others, spirituality, housing security, and access to medical services (Stephenson et al., 2023; Stroem et al., 2021; Valido et al., 2021).

In the context of climate-related crises, strengthening these protective factors becomes even more critical, as stable housing, family support, and access to medical care can buffer against the compounding effects of environmental stress and violence victimization. Substance use prevention may serve as an additional protective factor against violence victimization (Stroem et al., 2021). The impact of sociodemographic factors and other identified risk factors can partly moderate the relationship between IPV victimization and sexual identity (Hazelwood, 2023). Enhancing services and comprehensively addressing structural and intrapersonal factors could contribute to the prevention of IPV among transgender youth (Goldenberg et al., 2018; Levine and Button, 2022). In times of climate disruption, ensuring the continuity of services and access to supportive networks will be key to preventing further victimization among marginalized youth, including those in the 2SLGBTQIA+ community.

One study suggests that adopting a gender-inclusive approach could help prevent the commercial sexual exploitation of youth (COMSEY) (Shilo et al., 2021). Furthermore, 2SLGBTQIA+-affirming schools are associated with lower rates of physical IPV among female students (Adams et al., 2020). Similarly, implementing appropriate policies and practices regarding individual attitudes in schools may help prevent bullying perpetration (Chan and Lam, 2023; Murchison et al., 2023). As schools face the challenges of climate change, creating inclusive and supportive environments becomes even more important to reduce bullying and other forms of violence against 2SLGBTQIA+ youth.

### 3.4 Theme 4: experiences, perception and prevention of various types of violence, and help-seeking behaviors

2SLGBTQIA+ youth are consistently subjected to various forms of violence, including physical, emotional, verbal, sexual, and psychological violence, all of which negatively affect their lives (Brewer et al., 2024; Caputi et al., 2020; Dank et al., 2014; Fish et al., 2023; Nath et al., 2022; Whitton et al., 2019a,b; Wright et al., 2023). Some victims have reported experiencing two or more types of victimization multiple times (Price et al., 2023; Scheer et al., 2021; Stroem et al., 2021). In the context of climate change, these vulnerabilities may be exacerbated as environmental crises, such as displacement and resource scarcity, increase the risks of violence and victimization for 2SLGBTQIA+ youth, especially those who are marginalized or lack stable housing. 2SLGBTQIA+ youth face both GBV and sexuality-based violence, which occurs both in-person and online through comments, text messages, and phone calls (Gámez-Guadix and Incera, 2021; Mitchell et al., 2014; Taylor et al., 2021; Whitton et al., 2019a,b; Wright et al., 2023). Perpetrators of violence against 2SLGBTQIA+ youth often include family members, intimate partners, peers, teachers, and school staff (Aguilera-Jiménez et al., 2023; Brewer et al., 2024; Domínguez-Martínez et al., 2023; Kaufman and Baams, 2022; Stroem et al., 2021). Physical violence is more frequently inflicted by family members, while sexual violence is typically perpetrated by individuals outside the family (Strauss et al., 2020). In times of climate-induced stress and displacement, family dynamics may become strained, further increasing the likelihood of family-based violence, while the lack of safety in temporary shelters or unstable living situations may elevate the risk of sexual violence from external perpetrators. Additionally, in the context of relationship violence, the perpetrator's gender cannot be assumed based solely on a youth's sexual or gender identity, as both boys and girls can be perpetrators (Stroem et al., 2021).

Multiple studies have explored how 2SLGBTQIA+ youth perceive their experiences with violence. The majority of youth believe that sexual violence predominantly affects 2SLGBTQIA+ individuals and is perpetrated by heterosexual people (Nath et al., 2022). They feel targeted because of their sexual and gender identities, and many believe that their physical appearance, especially when their gender expression deviates from cis-normative expectations, makes them more vulnerable to sexual violence (Nath et al., 2022). As climate change disrupts communities, marginalized youth may become even more visible targets for violence due to the increased instability and breakdown of social structures, heightening their vulnerability to both sexual and physical violence. Research by Strauss et al. (2020) revealed that 2SLGBTQIA+ youth have varied views on the connection between their victimization and their transgender identity. They associate physical violence occurring outside the family with their trans identity but rarely link sexual violence to it. When it comes to other forms of violence, such as emotional or verbal abuse and neglect, some youth connect these experiences to their trans identity, while others believe the relationship is partial or situational (Strauss et al., 2020).

Martin-Storey et al. (2022) proposed that 2SLGBTQIA+ students' discussions of sexuality and gender in relation to their experiences of sexual violence highlight two key factors: their certainty about others' awareness of their identities and the time elapsed between disclosure and the violence. In the wake of climate-related crises, the likelihood of disclosure and seeking support may decrease as victims face new challenges such as displacement, loss of privacy, or fear of further stigmatization in unstable environments. They argue that individuals are more likely to link their 2SLGBTQIA+ identities to experiences of sexual violence when they are confident that the perpetrator was aware of this identity and when less time has passed since the disclosure (Martin-Storey et al., 2022).

A portion of the literature has examined 2SLGBTQIA+ youth's reactions to experiences of violence and their help-seeking behaviors. In documenting how 2SLGBTQIA+ youth respond to sexual violence, Martin-Storey et al. (2022) identified “pushout” as the most frequently reported reaction, manifesting in both social contexts (such as avoiding activities or withdrawing from school) and school settings (like transferring to a different school). Minimization was also observed as a common response to sexual violence, where individuals either downplayed the severity of their experiences or suggested that they did not face significant consequences from the incident (Martin-Storey et al., 2022). In the context of environmental crises, the additional stress and uncertainty may lead to even more youth withdrawing from school or social activities, further isolating themselves and minimizing their victimization as a coping mechanism.

The high rates of violence are concerning, particularly in light of research showing that most 2SLGBTQIA+ youth who experience violence do not seek help (Kaufman and Baams, 2022; Scheer et al., 2023; Wright et al., 2023). Climate change may further impede help-seeking behaviors, as displaced youth may lack access to trusted support networks or face heightened stigma in new environments. Barriers to seeking help for these victims include uncertainty about whom to approach, fear of reporting, reluctance to reinforce negative stereotypes about the 2SLGBTQIA+ community, a desire to keep their 2SLGBTQIA+ status private, and the belief that there are insufficient tailored services available (Kaufman and Baams, 2022; Scheer et al., 2023). Conversely, factors that facilitate help-seeking include interpersonal closeness, confidentiality, and affirmation of their 2SLGBTQIA+ identity (Scheer et al., 2023). The most common reason young victims sought help was the presence of a trusted person or provider who was aware of their abusive situation and would maintain the confidentiality of their disclosure (Scheer et al., 2023).

Among those who sought help, many did so only after experiencing multiple incidents of violence (Wright et al., 2023). When considering whom to approach for support, one study found that 2SLGBTQIA+ youth often turned to friends or chosen family members, while another indicated that they reported incidents to the police more frequently than to family members (Kaufman and Baams, 2022; Wright et al., 2023). However, in the context of climate change, where youth may be displaced or disconnected from their usual support networks, their help-seeking patterns may change, with fewer opportunities to turn to trusted friends or family members. 2SLGBTQIA+ youth reported facing specific pressures as victims when seeking support, particularly fears of being outed or stigmatized during the process (Nath et al., 2022). They also voiced concerns that 2SLGBTQIA+ individuals might be treated unfairly if they reported incidents of sexual violence to authorities. Additionally, among those who reported victimization, 2SLGBTQIA+ victims were less likely to see any action taken or have the issue resolved compared to their heterosexual, cisgender counterparts (Kaufman and Baams, 2022).

The reviewed literature has also focused on prevention and intervention strategies to mitigate victimization risks for 2SLGBTQIA+ youth. In the face of climate change, these strategies must also account for the additional vulnerabilities created by environmental crises, such as displacement and loss of social support. Some scholars are dedicated to engaging 2SLGBTQIA+ youth and incorporating their perspectives on how to prevent and address violence (e.g., Adhia et al., 2024; Burkholder et al., 2023; Coulter and Gartner, 2023). Adhia et al. (2024) investigated 2SLGBTQIA+ youth views on improving middle and high school environments to better prevent and address sexual violence. Youth identified several key elements for schools, including: (1) access to gender-neutral spaces; (2) 2SLGBTQIA+ competency training for staff; (3) enforcement of school policies related to sexual violence and anti-bullying, along with accountability; (4) 2SLGBTQIA+-competent mental health support; and (5) comprehensive sexual health education that includes 2SLGBTQIA+ relationships and sexual violence (Adhia et al., 2024). As climate change disrupts communities, the creation of safe, inclusive school environments becomes even more crucial to protecting marginalized youth from violence. Coulter and Gartner (2023) adopted a youth-centric approach to collaborate with 2SLGBTQIA+ youth in developing intervention concepts aimed at reducing dating violence, resulting in eight intervention strategies to improve education, support systems, and advocacy efforts. Burkholder et al. (2023) also involved queer, trans, and non-binary youth in a participatory visual research project, which facilitated media co-production and provided a platform for showcasing queer maker literacies while fostering solidarity, action, and safety.

Some researchers have focused on developing new prevention strategies, while others have evaluated or modified existing violence prevention programs (e.g., Coker et al., 2020; Crooks et al., 2018; Tam and Brown, 2020; Wesche et al., 2021; Lessard et al., 2020; Kaltiala and Ellonen, 2022). Wesche et al. (2021) adapted the evidence-based Safe Dates dating violence prevention program to include 2SLGBTQIA+ youth, addressing specific risk factors relevant to this group. As environmental stressors increase, expanding such programs to address the compounded risks of climate change will be essential to safeguarding 2SLGBTQIA+ youth. They implemented the expanded curriculum, serving both 2SLGBTQIA+ and cisgender, heterosexual youth. Initial results indicated that the program was feasible and effective, significantly enhancing participants' knowledge about dating violence. Another study used a rigorous cluster randomized control trial to assess the effectiveness of bystander interventions designed to reduce sexual violence and harassment among both sexual majority and 2SLGBTQIA+ high school youth (Coker et al., 2020). This intervention resulted in decreased stalking victimization and perpetration among 2SLGBTQIA+ youth (Coker et al., 2020). As climate change continues to disrupt social and educational systems, bystander interventions and inclusive violence prevention programs will be vital in mitigating the compounded risks faced by 2SLGBTQIA+ youth.

### 3.5 Theme 5: differences among subgroups of 2SLGBTQIA+ youth in violence perpetration and victimization

Victimization experiences are disproportionately prevalent among subpopulations of 2SLGBTQIA+ youth (Kosciw et al., 2012). Female-identifying youth consistently face a greater risk of physical and sexual violence compared to male youth (Strauss et al., 2020; Taylor et al., 2021; Whitton et al., 2019a; Williams and Gutierrez, 2022). In the context of climate change, these risks may be exacerbated, as environmental crises can lead to displacement, resource scarcity, and increased vulnerability, particularly for female-identifying youth who may lack access to secure housing or protective services during disasters.

When examining the intersection of gender and sexual orientation, 2SLGBTQIA+ females have the highest likelihood of experiencing dating and sexual violence victimization, followed by 2SLGBTQIA+ males and heterosexual females (Semprevivo, 2021). In times of environmental crises, these disparities may become more pronounced, as 2SLGBTQIA+ youth groups, particularly bisexual and uncertain youth, face increased isolation and vulnerability during displacement or resource scarcity. Indeed, disparities in victimization rates exist between sexes and subgroups of 2SLGBTQIA+ youth. For instance, bisexual female youth are particularly at risk compared to those identifying as heterosexual, lesbian, or unsure of their sexual identity (Johns et al., 2018; Chmielewski, 2017). Among males, those uncertain about their sexual identity report a greater prevalence and frequency of dating violence victimization than their heterosexual or even 2SLGBTQIA+ peers (Olsen et al., 2020; Heino et al., 2020).

For transgender youth, GBV risk is often linked to societal stigma and discrimination surrounding gender identity. Transgender youth frequently face targeted violence, including physical assaults and sexual violence, particularly in public spaces, schools, or while accessing healthcare. This heightened vulnerability is often exacerbated by systemic barriers, such as the lack of access to gender-affirming medical care and legal recognition of their gender identity. These barriers not only reinforce marginalization but can also expose transgender youth to additional violence when forced to interact with systems or environments that disregard their identities. Transgender youth who are visibly gender-nonconforming are at a higher risk of violence, as their appearance or behavior may challenge societal norms more overtly, making them more susceptible to hostility.

Non-binary youth, while sharing some risks with transgender youth, face unique challenges due to their position outside the traditional gender binary. Non-binary identities are often less visible or understood, leading to erasure and invalidation in social, legal, and institutional contexts. This invisibility can compound their vulnerability to GBV, as non-binary youth may struggle to find spaces or resources specifically designed to support their needs. Additionally, the ambiguity surrounding non-binary identities in many societal frameworks often results in misgendering, harassment, and exclusion, further increasing their exposure to violence. Non-binary youth may also experience unique forms of GBV that are tied to their gender expression, such as being coerced to conform to binary gender norms through verbal abuse or forced assimilation.

2SLGBTQIA+ youth not only experience higher rates of victimization, but they are also targeted by different perpetrators, such as teachers and other school staff, and in more private settings (Kaufman and Baams, 2022). Their victimization is less likely to be acknowledged or addressed by those responsible for their safety, including teachers and parents (Kaufman and Baams, 2022). In the context of climate-related disruptions, where school and home environments may become destabilized, the risks of unacknowledged or unaddressed victimization could rise, leaving 2SLGBTQIA+ youth even more vulnerable to ongoing violence. 2SLGBTQIA+ youth reported receiving less social support from family and significant others compared to their heterosexual, cisgender peers, and a lack of understanding of violence within the 2SLGBTQIA+ community (Nath et al., 2022; Sabina et al., 2022). For individuals identified as 2SLGBTQIA+, family support was noted as a protective factor against depression, peer victimization, bullying, and sexual perpetration (Mintz et al., 2021). As climate change impacts social and family dynamics, maintaining strong support networks will be crucial in mitigating the mental health impacts of victimization among these youth.

Moreover, transgender and gender-nonconforming youth are even more likely to encounter severe sexual harassment than their male counterparts (Mitchell et al., 2014). Transgender youth report the highest rates of sexual harassment, followed by gender-nonconforming and other 2SLGBTQIA+ youth (Mitchell et al., 2014). Additionally, 2SLGBTQIA+ youth, regardless of their sex assigned at birth, report elevated rates of poly-victimization (Scheer et al., 2021). Climate-induced stressors, such as displacement and loss of social networks, may further increase the risk of poly-victimization for these vulnerable subpopulations. Furthermore, transgender and nonbinary youth experience higher rates of physical dating violence victimization compared to cisgender 2SLGBTQIA+ youth (Price et al., 2023). Bisexual or questioning youth are more likely to experience sexual victimization compared to their gay or lesbian counterparts (Whitton et al., 2019a). A similar conclusion was reached in another study, which found that bisexual youth report higher rates of dating violence, sexual assault, and overall dating experiences than their lesbian and gay peers (Kiekens et al., 2022). As climate change disrupts social and community structures, bisexual and questioning youth may face additional challenges in seeking support or escaping violent situations, further heightening their vulnerability to sexual victimization.

Risk levels also vary with age. Younger 2SLGBTQIA+ youth are more likely to report experiences of sexual harassment, 2SLGBTQIA+-based bullying, and poly-victimization compared to their older counterparts (Scheer et al., 2021). In the context of environmental crises, younger youth may be particularly vulnerable, as they often rely more heavily on family or school support systems, which may be disrupted during displacement or disaster scenarios. The odds of sexual victimization increase more with age for male 2SLGBTQIA+ youth than for female 2SLGBTQIA+ youth (Whitton et al., 2019a). Among a sample of transgender youth, those aged 11 and younger, as well as those aged 18 and older, report significantly higher rates of bias-based bullying (Fish et al., 2023).

Patterns of violence victimization also differ among racial and ethnic groups. While White and Asian youth have a lower likelihood of experiencing forced sexual intercourse and sexual violence, Black youth are less likely to experience sexual dating violence (Williams and Gutierrez, 2022). The rate of physical victimization is higher for racial-ethnic minorities than for White youth (Whitton et al., 2019a). In times of climate-induced instability, racial and ethnic minority youth may be further marginalized, facing heightened barriers to safety and protection, leading to greater exposure to violence, particularly physical and sexual victimization. Other research also indicates that racial minority youth are more likely to experience higher rates of most IPV types (Whitton et al., 2023) and partner victimization compared to their White counterparts (Walls et al., 2019; Whitton et al., 2019b). Additionally, while the prevalence of physical IPV decreases with age among White youth, it remains stable for racial-ethnic minorities (Whitton et al., 2019a). A study on Indigenous 2SLGBTQIA+ young people highlights substantial instances of family and community violence, characterized by intimidation, bullying, and threats (Soldatic et al., 2023). Some of the violence is linked to their gender and sexual diversity, while other forms of victimization are connected to perceptions of their indigeneity. As climate change disproportionately affects Indigenous communities, the compounded risks of gender and racial-based violence in these contexts are likely to increase, necessitating targeted interventions that address both environmental and social vulnerabilities.

Overall, 2SLGBTQIA+ youth with intersecting socially marginalized identities—such as being transgender, multisexual, Black, Native/Indigenous, Latinx, or multiracial; experiencing economic hardship or homelessness; and identifying as a feminine person assigned male at birth, a masculine person assigned female at birth, or a nonbinary person expressing high masculinity or femininity—are particularly vulnerable to victimization (Price et al., 2023). In the context of climate change, these intersecting identities may further compound the challenges these youth face, as marginalized individuals are often disproportionately affected by environmental crises and have fewer resources to escape or recover from victimization. For instance, when examining rates of sexual harassment, lesbian and queer girls report the highest levels across modes and types, followed by bisexual girls and gay/queer boys, while heterosexual boys report the lowest rate (Mitchell et al., 2014). Addressing the needs of these highly vulnerable subpopulations requires comprehensive strategies that take into account both the social and environmental factors contributing to their victimization.

## 4 Strengths

This literature review significantly enriches the understanding of GBV among 2SLGBTQIA+ youth by providing a comprehensive examination of various forms of victimization and their associated mental health outcomes, particularly in the context of climate change. By covering intimate partner violence, bullying, harassment, and other types of violence, the review offers a holistic perspective on the risks faced by 2SLGBTQIA+ youth, who are disproportionately affected by depression, anxiety, suicidality, and substance use—risks that are often exacerbated by climate-related stressors such as displacement, housing instability, and disrupted social and educational environments. Additionally, the analysis of protective factors, such as family support and school belonging, provides critical insights into mitigating negative mental health impacts, especially during times of crisis when support systems may be weakened or inaccessible.

These findings highlight not only the risk factors but also the potential for resilience, presenting a balanced view that can inform targeted intervention strategies adapted to climate-induced vulnerabilities. The comparative approach between 2SLGBTQIA+ and non-2SLGBTQIA+ youth adds depth to the discussion by illustrating the unique disparities in victimization rates, which can be magnified by inequities in disaster responses and recovery efforts. The review's focus on subgroups, including bisexual and transgender youth, uncovers variations in experiences that necessitate tailored support, particularly as these groups may face heightened barriers in accessing inclusive emergency services and safe housing during climate crises. Furthermore, the examination of help-seeking behaviors emphasizes the barriers 2SLGBTQIA+ youth face, such as stigma and inadequate services, while also pointing to factors that facilitate support, like confidentiality and trusted relationships. By evaluating existing prevention programs and recommending culturally competent, 2SLGBTQIA+-affirming interventions, the review provides evidence-based strategies for enhancing support systems and reducing GBV among 2SLGBTQIA+ youth, with a lens toward addressing vulnerabilities amplified by climate change.

## 5 Limitations

This review provides insights into the experiences of 2SLGBTQIA+ youth facing GBV in the context of climate change but has several limitations. A major limitation is its reliance on English-language studies, excluding research from regions like Latin America, Africa, and Asia, where climate-related disasters and vulnerabilities are most severe. This exclusion limits understanding of diverse cultural and social contexts.

Geographic representation is another concern, with most studies focusing on high-income countries, especially the U.S., whose social, legal, and healthcare systems differ from those in low- and middle-income countries. As a result, the findings may not fully reflect the experiences of 2SLGBTQIA+ youth globally, particularly in regions with higher stigma or fewer protections.

Inconsistencies in how GBV is defined and measured also pose challenges, with some studies focusing narrowly on physical violence and others adopting broader definitions. This variability complicates synthesis and underscores the need for standardized methodologies in future research.

The review highlights gaps in representation of subgroups like Indigenous youth, youth of color, and those in rural areas, whose intersecting vulnerabilities are underexplored. This limitation emphasizes the importance of intersectional approaches to better inform inclusive policies and interventions.

Variability in methodological quality, including small sample sizes and limited intersectional analyses, weakens the evidence base. Temporal limitations also affect the review, as older studies may not reflect current realities, while newer ones may lack long-term data on climate impacts.

Finally, the influence of political and legal contexts on GBV and climate change research cannot be overlooked. Restrictive laws and policies in some regions increase risks for 2SLGBTQIA+ youth, while progressive frameworks elsewhere reduce vulnerabilities. Future studies must address these complexities to better inform global policies and interventions.

## 6 Implications

The findings from this review highlight critical vulnerabilities among 2SLGBTQIA+ youth to GBV and adverse mental health outcomes, compounded by the stressors of climate change. These insights necessitate targeted policy interventions, evidence-based research, and inclusive social work practices to mitigate risks and improve outcomes for this population.

### 6.1 Policy implications

Policymakers should prioritize inclusive, intersectional frameworks to address GBV against 2SLGBTQIA+ youth by mandating gender-affirming healthcare, safe housing, anti-discrimination protections, and explicit anti-bullying laws with enforcement mechanisms. Disaster response and recovery policies should ensure access to safe shelters, mental health services, and gender-affirming care, while emergency preparedness frameworks must include measures such as 2SLGBTQIA+ training for responders, gender-neutral shelter spaces, and tailored resources. Additionally, international climate migration policies should recognize the compounded vulnerabilities of 2SLGBTQIA+ individuals, offering legal protections and pathways to safety and support. Programs such as microfinance interventions, which have proven effective in empowering women and reducing and reducing GBV in other contexts (Sabri et al., 2023), highlight the potential for economic initiatives tailored to 2SLGBTQIA+ communities in climate-affected regions.

### 6.2 Research implications

The reviewed literature highlights the need for inclusive, geographically diverse research to capture the lived experiences of 2SLGBTQIA+ youth, emphasizing the intersection of climate change, GBV, and mental health. Future studies should explore how environmental crises exacerbate vulnerabilities, with longitudinal research examining the long-term impacts of displacement and resource scarcity. Attention must also focus on subgroups like transgender, non-binary, and bisexual youth to identify specific risks and protective factors, informing tailored interventions. Participatory methodologies engaging 2SLGBTQIA+ youth as co-researchers can provide valuable insights while empowering them to shape solutions.

### 6.3 Social work practice implications

Social workers play a vital role in supporting 2SLGBTQIA+ youth affected by GBV and climate-related stressors by adopting trauma-informed, intersectional approaches that recognize their unique vulnerabilities and strengths. Interventions should build resilience through protective factors like family support, school belonging, and mental health resources while promoting inclusive environments via staff training, anti-violence policies, and gender-neutral facilities. In climate-affected areas, social workers must advocate for the inclusion of 2SLGBTQIA+ youth in disaster preparedness and recovery efforts. Additionally, they should champion systemic changes, such as policies protecting against GBV, expanding gender-affirming care, and addressing climate vulnerabilities, while connecting youth to resources like safe housing and legal assistance to foster resilience and wellbeing.

## 7 Conclusion

This literature review has underscored the significant and disproportionate risks of GBV faced by 2SLGBTQIA+ youth, along with the associated adverse mental health outcomes. The elevated rates of victimization, including intimate partner violence, bullying, and harassment, reveal persistent vulnerabilities within this population, leading to increased risks of depression, anxiety, suicidal ideation, and substance use. The review also highlights the critical role of protective factors such as family support, school belonging, and social networks in mitigating these negative effects, suggesting pathways for intervention that can enhance resilience and wellbeing among 2SLGBTQIA+ youth.

Moreover, the findings emphasize the need to integrate considerations of climate change and climate-induced disasters into GBV prevention strategies, as these emerging challenges can exacerbate existing risks for 2SLGBTQIA+ youth. Addressing the compounded vulnerabilities requires multi-faceted and intersectional approaches that encompass individual, familial, and structural interventions. By aligning GBV prevention with climate adaptation efforts and prioritizing the safety of marginalized groups in emergency response planning, it is possible to build more resilient and inclusive communities. Future research should aim to expand beyond U.S.-centric studies, incorporating diverse cultural and geographic perspectives to develop comprehensive strategies that address the unique needs of 2SLGBTQIA+ youth worldwide.

## Data Availability

The datasets presented in this study can be found in online repositories. The names of the repository/repositories and accession number(s) can be found at: https://doi.org/10.17605/OSF.IO/JC94X.

## Footnotes

^1^We use 2SLGBTQIA+ as an umbrella term to describe people who are sexual minorities (e.g., Two-Spirit, lesbian, gay, bisexual, transgender, queer, intersex, and additional people who identify as part of sexual and gender diverse communities) and gender minorities (e.g., transgender, genderqueer, and nonbinary).
